# Conserved minimal CD8 cytotoxic immune-state unit in an IL18R1-associated immune program in hepatocellular carcinoma

**DOI:** 10.1007/s00262-026-04487-9

**Published:** 2026-07-06

**Authors:** Masamichi Kimura, Koji Nishikawa, Jun Imamura, Kiminori Kimura

**Affiliations:** Department of Hepatology, Tokyo Metropolitan Cancer and Infectious Diseases Center, Komagome Hospital, 3-18-22 Honkomagome, Bunkyo-Ku, Tokyo, 113-8677 Japan

**Keywords:** Hepatocellular carcinoma, Tumor microenvironment, CD8 + T cells, IL-18, IL18R1, Single-cell transcriptomics

## Abstract

**Background and aims:**

The conserved human correlate of interleukin-18 receptor 1 (IL-18R1)-associated immune states in hepatocellular carcinoma (HCC) remains undefined. We aimed to identify a minimal, reproducible, immune-state component associated with IL18R1 across independent human HCC cohorts and evaluate its clinical relevance.

**Methods:**

Publicly available single-cell RNA sequencing datasets from five independent HCC cohorts were analyzed using a unified framework. Paired tumor-adjacent cohorts were prioritized for primary analyses, whereas tumor-only cohorts served as supportive datasets. CD8 T cells were stratified by IL18R1 expression, and tumor-specific enrichment was quantified using difference-in-differences. We validated the spatial transcriptomics using a murine liver tumor Xenium dataset. Clinical relevance was evaluated using The Cancer Genome Atlas Liver Hepatocellular Carcinoma (TCGA-LIHC) bulk RNA-seq data.

**Results:**

IL18R1-positive CD8 T cells exhibited a tumor-specific, C–C motif chemokine ligand 5 (CCL5)-centered cytotoxic transcriptional program, consistently enriched across datasets. Alternative IL18R1 stratification confirmed the identified immune program’s robustness. Spatial transcriptomics localized this cytotoxic state in tumor-associated CD8 T cells and highlighted increased expression of Ccl5 and Cxcr3. Tumor-only cohorts demonstrated substantial heterogeneity, highlighting the importance of paired cohort design. In TCGA-LIHC, a composite CCL5-centered cytotoxic core score, not CCL5 alone, was associated with improved overall survival, although this attenuated after adjusting for clinical covariates.

**Conclusions:**

The dominant human correlate of an IL18R1-associated immune state in HCC is a compact, CCL5-centered CD8 cytotoxic module, not a broad inflammatory response. This spatially validated unit provides a reproducible framework for HCC immune-state characterization and stratification.

**Supplementary Information:**

The online version contains supplementary material available at https://doi.org/10.1007/s00262-026-04487-9.

## Introduction

Hepatocellular carcinoma (HCC) arises within a complex immune microenvironment in which immune cell composition, density, and functional states substantially influence tumor progression and clinical outcomes [1, 2]. Hepatocarcinogenesis develops in the setting of chronic liver inflammation, creating a dynamic interplay between tumor-promoting and tumor-restraining immune responses [3]. Consequently, identifying immune programs associated with effective antitumor activity remains a major challenge in HCC research, particularly the identification of stable and reproducible immune-state units that can be translated across independent studies and clinical settings.

HCC remains a major global health burden and is one of the leading causes of cancer-related mortality worldwide, accounting for more than 800,000 deaths annually [4]. Despite advances in surveillance, systemic therapies, and immunotherapy, clinical outcomes remain highly variable among patients. This variability is partly driven by the substantial molecular, cellular, and immune heterogeneity that characterizes HCC across patients and within individual tumors [5]. Consequently, identifying conserved immune programs that are reproducible across independent cohorts has become an important objective in translational immuno-oncology research.

Single-cell transcriptomic technologies provide the cellular resolution necessary to dissect this heterogeneity and to identify conserved immune-state modules that are robust across independent cohorts. Such conserved immune programs may offer more biologically robust insights than individual genes or cohort-specific signatures and may facilitate immune-state characterization for immune stratification [6].

Recent advances in single-cell RNA sequencing have revealed diverse T cell states within the HCC immune microenvironment, including cytotoxic, exhausted, regulatory, and memory-like populations [6]. Although immune-active subsets characterized by cytolytic and inflammatory transcriptional programs have been described [7], these states often vary across datasets, patients, and tumor regions. Ecosystem-level analyses have further demonstrated close relationships between tumor cell diversity and immune composition [8], emphasizing the complexity of immune heterogeneity in HCC. However, the reproducible identification of conserved immune programs across independent human cohorts remains unresolved.

Interleukin-18 (IL-18), a member of the IL-1 cytokine family, is a potent regulator of type 1 immunity and plays a central role in the activation of cytotoxic lymphocytes. IL-18 promotes interferon-gamma (IFNG) production, enhances natural killer (NK) cell function, and cooperates with other cytokines to augment CD8 + T cell effector responses. In HCC, both experimental and clinical studies have implicated IL-18 signaling in shaping antitumor immunity, although its effects appear context-dependent and may vary according to the composition of the tumor microenvironment. Recent interest in IL-18-based immunotherapies has further highlighted the need to define the immune programs associated with IL18R1 expression in tumor-infiltrating lymphocytes. Mechanistically, IL-18 signals through the IL18R1–IL18 receptor accessory protein (IL18RAP) receptor complex via nuclear factor kappa-light-chain-enhancer of activated B cells and mitogen-activated protein kinase pathways, cooperating with IL-12/signal transducer and activator of transcription 4 (STAT4)-driven programs to enhance CD8 + T cell cytotoxic responses [9, 10].

Despite these observations, the transcriptional programs associated with IL18R1 expression in tumor-infiltrating CD8 + T cells remain incompletely characterized in human HCC, particularly the IL-18-associated components that are consistently preserved across independent datasets. Evidence suggests that tumor-infiltrating T cells exist along a continuum of overlapping transcriptional states shaped by multiple biological factors [11, 12]. This raises the question: which elements of an IL18R1-associated immune state are biologically stable, reproducible, and spatially conserved across independent human cohorts.

A major challenge in analyzing immune states using public datasets is study design. Many available cohorts are tumor-only and cross-sectional, making them vulnerable to interpatient variability and cohort-specific effects. In contrast, datasets containing paired tumor and adjacent liver samples permit within-patient comparisons and provide a more robust framework for identifying tumor-specific immune programs. This consideration is particularly relevant for chemokine-associated immune states because lymphocyte recruitment and positioning are highly dependent on tissue context. CCL5-centered programs are of particular interest because they have been implicated in T cell recruitment, cytotoxic immune activity, and antitumor responses [13]. In a recent spatial transcriptomic study of murine liver tumors, we identified an IL18R1-associated immune state linked to cytotoxic lymphocyte activation and enrichment of effector-associated genes [14]. However, the reproducibility and biological relevance of this immune state in human HCC remain unclear. Specifically, it remains unknown whether cytotoxic, inflammatory, or memory-associated components represent the most conserved element of the IL18R1-associated immune program across independent datasets.

In this study, we aimed to define the conserved human correlate of an IL18R1-associated immune state by analyzing multiple public single-cell RNA sequencing datasets of HCC using a unified analytical framework. We prioritized cohorts with paired tumor and adjacent liver samples and applied patient-level analyses to assess reproducibility across studies. We found that the most consistently preserved component of the IL18R1-associated immune program was a compact CD8 cytotoxic module enriched for CCL5 and effector genes, whereas broader inflammatory programs were less reproducibly conserved. We further evaluated the robustness of this immune-state unit using alternative stratification strategies, spatial transcriptomic analyses, and independent clinical datasets. Our findings highlight the importance of paired cohort designs and spatial validation for identifying conserved immune programs in HCC and provide a biologically interpretable framework for immune-state characterization across independent datasets.

## Materials and methods

### Study design

This study aimed to identify the conserved human correlate of an IL18R1-associated immune state in HCC by analyzing publicly available single-cell RNA sequencing datasets using a unified analytical framework. We prioritized datasets with paired tumor and adjacent liver samples for primary analyses due to substantial interpatient heterogeneity in tumor-only datasets. Tumor-only cohorts were analyzed as supportive datasets to assess the robustness of the identified immune programs across independent studies.

Immune populations were extracted across datasets, a broad CD8-like compartment was identified based on canonical T cell marker expression, and cells were stratified by IL18R1 expression status. Sample-level pseudobulk analyses were performed to enable cohort-level comparisons while reducing noise in single-cell measurements. In addition to public human datasets, spatial transcriptomic analyses were conducted using a previously generated Xenium dataset from a murine liver tumor model for orthogonal validation of the identified immune state at single-cell and spatial resolution.

### Public single-cell datasets

Publicly available human HCC single-cell RNA sequencing datasets were obtained from the Gene Expression Omnibus (GEO). The final analyses included five datasets: GSE189903, GSE149614, GSE140228, GSE151530, and GSE125449. Datasets with paired tumor and adjacent liver samples were used for primary analyses, whereas tumor-only cohorts served as supportive datasets. Raw gene expression matrices and associated metadata were downloaded from GEO and processed independently using a standardized analysis pipeline. A summary of cohort characteristics and the number of evaluable patients included in each analysis is provided in Supplementary Table S1.

### Dataset selection rationale

Public datasets were selected based on predefined criteria to enable the reproducible evaluation of IL18R1-associated immune states in human HCC. First, datasets were required to contain human HCC samples with available single-cell transcriptomics data. Second, datasets that included paired tumor and adjacent liver samples were prioritized to allow within-patient comparisons and reduce interpatient heterogeneity. Third, dataset inclusion required sufficient immune cell representation to identify CD8 + T cell populations and evaluate IL18R1-associated transcriptional programs.

Based on these criteria, three paired datasets (GSE189903, GSE149614, and GSE140228) were selected as primary cohorts, and two tumor-only datasets (GSE151530 and GSE125449) were included as supportive cohorts.

### Data preprocessing

Gene expression matrices were processed in R (version 4.4.3) using Seurat (version 5.4.0). Gene symbols were standardized across datasets for cross-cohort comparisons. Quality control procedures were applied based on dataset characteristics. Cells with extremely low detected genes or abnormally high transcript counts were excluded to remove low-quality cells or potential multiplets. For large datasets where loading the full count matrix exceeded available memory, matrix subsetting was performed prior to downstream analyses to focus on immune populations of interest.

Immune compartments were identified using annotation metadata from the original studies. When necessary, additional marker-based filtering was applied to harmonize immune cell definitions across cohorts.

### Identification of CD8 T cell populations

For each dataset, lymphoid populations were identified according to the original cell annotations provided by the corresponding study. To maximize consistency across cohorts while preserving evaluable cell numbers, primary analyses were performed on a broad CD8-like compartment defined by canonical T cell markers (CD3D, CD3E, and/or CD3G) together with CD8A and/or CD8B. Cells lacking T cell lineage markers were excluded. Because cytotoxic lymphocytes may exhibit overlapping transcriptional programs, additional robustness analyses were performed using stricter CD8 T cell definitions that required stronger T cell lineage annotation and exclusion of NK-like populations when dataset-specific annotations permitted. Results obtained using broad and strict CD8 T cell definitions were compared to assess the robustness of identified immune programs.

Within each dataset, cells in the broad CD8-like compartment were stratified according to IL18R1 expression. Because IL18R1 transcripts were sparsely detected in several cohorts, the primary analysis classified cells as IL18R1-positive (detectable expression) or IL18R1-negative (undetectable expression). To evaluate the robustness of this approach, additional analyses were performed using alternative IL18R1 stratification strategies, including quartile-based comparisons among IL18R1-positive cells. Consistent enrichment patterns across alternative stratification methods demonstrated robustness.

For patient-level analyses, pseudobulk profiles were generated by averaging normalized expression values within each patient, tissue compartment, and IL18R1-defined subgroup. These pseudobulk profiles were subsequently used for signature-score calculation and cohort-level comparisons.

### IL18R1 expression stratification

Cells within the broad CD8-like compartment were stratified into IL18R1-positive and IL18R1-negative populations based on detectable IL18R1 expression. A binary threshold (nonzero vs. zero expression) was applied to ensure reproducibility across cohorts owing to the sparse detection of IL18R1 transcripts in single-cell datasets. Minimum cell thresholds were applied to ensure stable pseudobulk estimation because IL18R1 transcripts were sparsely detected in several datasets.

To evaluate whether this binary stratification was influenced by transcript detection depth, additional analyses were performed in the Xenium spatial transcriptomic dataset. Relationships between IL18R1 status and sequencing complexity metrics (nFeature_RNA and nCount_RNA) were examined, and logistic regression analyses incorporating nFeature_RNA as a covariate were conducted. These analyses were used to determine whether IL18R1-associated transcriptional programs remained detectable after adjustment for transcript detection depth. In addition, among IL18R1-positive CD8 + T cells in the Xenium dataset, alternative stratification analyses were performed using expression-based quartiles. IL18R1-low and IL18R1-high populations were defined as the lower and upper quartiles of IL18R1 expression, respectively, to assess whether observed transcriptional programs remained consistent under alternative thresholding strategies.

### Pseudobulk construction

Pseudobulk expression profiles were generated to reduce single-cell noise and enable sample-level comparison. Within each patient sample, CD8-like cells were separated by IL18R1 status, and gene counts were aggregated to create patient-level pseudobulk profiles. The aggregated matrices served as sample-level expression profiles.

Counts per million (CPM) values were calculated by dividing gene counts by the total library size of each pseudobulk sample and multiplying by one million. The expression values were transformed using log1p transformation. Signature scores were calculated based on these log1p(CPM) values.

Only samples with a minimum number of cells in IL18R1-positive and IL18R1-negative groups were included. Sensitivity analyses were conducted using minimum thresholds of 10, 5, and 3 cells. The threshold for paired analyses preserved evaluable paired samples while maintaining the stability of pseudobulk estimates. For tumor-only supportive analyses, the minimum threshold was selected to maximize the number of evaluable samples while retaining both IL18R1 strata within each sample.

### Gene signature analysis

To characterize the functional immune programs associated with IL18R1 expression, predefined gene signatures were evaluated.

The primary cytotoxic signature included genes associated with activated cytotoxic lymphocytes, including *CCL5*, perforin 1 (*PRF1*), granzyme B (*GZMB*), granzyme H (*GZMH*), granulysin (*GNLY*), natural killer cell granule protein 7 (*NKG7*), XC motif chemokine ligand 1 (*XCL1*), XC motif chemokine ligand 2 (*XCL2*), and beta-2 microglobulin (*B2M*), together with the activation-associated marker HLA class II histocompatibility antigen, DR alpha (*HLA-DRA*). This signature was designed to capture activated cytotoxic lymphocyte states rather than exclusively cytotoxic effector functions.

Secondary signatures representing effector-memory and IFNG-inducible inflammatory transcriptional programs were also evaluated. The effector-memory signature included *CCL5*, *NKG7*, *GNLY*, *PRF1*, *GZMB*, *GZMK*, CXC chemokine receptor 3 (*CXCR3*), and *IFNG*. The gene signature for IFNG-inducible inflammatory transcriptional program included *CCL5*, *IFNG*, *STAT1*, interferon regulatory factor 1 (*IRF1*), chemokine C-X-C motif ligand 9 (*CXCL9*), *CXCL10*, *CXCL11*, guanylate-binding protein 1 (*GBP1*), *GBP2*, and *B2M*.

Pseudobulk matrices were converted to CPM and log1p-transformed, and signature scores were calculated as the mean expression of genes within each signature. A complete summary of all gene signatures, constituent genes, and analytical applications is provided in Supplementary Table S2.

### Paired cohort analysis

Within-patient comparisons were performed for datasets containing paired tumor and adjacent liver samples to minimize interpatient variability. For each patient, the difference between IL18R1-positive and IL18R1-negative CD8-like pseudobulk signature scores was calculated separately for the tumor and adjacent liver. To assess whether IL18R1-associated programs were preferentially enriched in tumors relative to adjacent tissues, a difference-in-differences (DiD) framework was applied as follows:

DiD = (tumor IL18R1-positive − tumor IL18R1-negative) − (adjacent IL18R1-positive − adjacent IL18R1-negative)

This paired DiD design was chosen as the primary analytical framework because it controls for interpatient baseline immune variation and enables isolation of tumor-specific IL18R1-associated effects, representing a critical advantage over cross-sectional designs.

### Tumor-only cohort analysis

Datasets containing tumor samples without matched adjacent tissues were analyzed separately as supportive cohorts. In these cohorts, the IL18R1-positive and IL18R1-negative CD8-like fractions were compared within each tumor sample using the same pseudobulk framework. Due to the lack of paired adjacent controls, these analyses were interpreted as supportive validation of directional consistency rather than primary evidence. Exploratory robustness analyses of alternative transcriptional signatures, gating strategies, and tumor-only cohort evaluability were performed and are summarized in Supplementary Fig. S1.

### Statistical analysis

Comparisons between IL18R1-defined groups were performed using patient-level pseudobulk data. For each dataset, signature scores were calculated for individual patients, and differences between IL18R1-defined groups were quantified. Tumor-specific enrichment was assessed using a DiD framework that compared IL18R1-associated contrasts between tumor and adjacent liver tissues. Summary metrics included mean DiD values, median differences, and the proportion of patients demonstrating positive enrichment.

Because the primary objective of this study was to identify reproducible immune-state components across independent cohorts rather than to estimate a single pooled effect size, analyses emphasized directional consistency and effect-size concordance across datasets. Cohort-level mean DiD values and the proportion of patients showing positive enrichment were, therefore, reported as measures of reproducibility.

Nonparametric comparisons were performed using the Wilcoxon rank-sum test or Wilcoxon signed-rank test, as appropriate. Fisher’s exact test was used for categorical comparisons. Survival analyses were performed using Kaplan–Meier methods, log-rank tests, and Cox proportional hazards regression models. Multivariable Cox models included age and American Joint Committee on Cancer (AJCC) stage as covariates. Hazard ratios (HRs) and 95% confidence intervals (CIs) were reported where applicable.

To evaluate whether transcript detection depth acted as a confounder, additional analyses were performed in the Xenium dataset using nFeature_RNA-adjusted models. Statistical significance was defined as a two-sided *P* value < 0.05. All analyses were performed using reproducible scripts implemented in R.

### Xenium spatial transcriptomic analysis

To evaluate the biological and spatial relevance of the conserved IL18R1-associated immune program, a previously generated Xenium spatial transcriptomic dataset derived from a murine liver tumor model treated with IL-2, IL-18, and programmed death-ligand 1 (PD-L1) blockade was analyzed [14]. Preprocessed Xenium data, including cell-by-gene expression matrices and cell annotations, were analyzed using Seurat (version 5). Major cellular populations were identified through unsupervised clustering and marker-gene expression patterns.

To directly assess the relationship between IL18R1 expression and the conserved cytotoxic program, Xenium CD8 T cells were stratified according to IL18R1 expression status. Differential expression analyses were performed between IL18R1-high and IL18R1-low CD8 T cells within tumor regions. Additional analyses were conducted in treatment-associated tumor regions to evaluate the consistency of identified associations.

A CD8 cytotoxic core score was calculated at the single-cell level using a predefined gene set adapted to the Xenium panel (*CCL5, PRF1, GZMB, NKG7, GNLY, GZMK, XCL1, XCL2*, and *CXCR3*). Scores were compared across major cell populations, within CD8 T cells, and across spatial and experimental conditions. Spatial analyses focused on tumor versus non-tumor regions and control versus treatment groups.

For visualization purposes, representative spatial maps of Cd8a-positive cells, Ccl5-high CD8 T cells, and Gzmb-high CD8 T cells were generated from tumor regions. High-expressing populations were defined as the top 10% of CD8 T cells ranked according to gene expression. These visualizations were intended to provide representative spatial illustrations of the conserved cytotoxic program and were not used for statistical inference.

### The Cancer Genome Atlas Liver Hepatocellular Carcinoma (TCGA-LIHC) bulk transcriptomic and survival analysis

To evaluate the clinical relevance of the cytotoxic core, we analyzed bulk RNA sequencing and clinical data from the TCGA-LIHC cohort using TCGAbiolinks. Gene expression was based on STAR-count data, normalized with log2 CPM, and cytotoxic core scores were calculated as the mean z-scored expression of predefined genes.

A CCL5-centered cytotoxic core score was calculated for each sample as the mean expression of specified cytotoxic genes (*CCL5, GZMA, GZMB, PRF1, NKG7, IFNG, CXCL9,* and *CXCL10*). Patients were stratified into high and low groups based on the median core score, with consistent trends observed using alternative thresholds. Overall survival was assessed between groups using Kaplan–Meier analysis and the log-rank test.

### Computational environment

All analyses were performed using R (version 4.4.3). Single-cell analyses were conducted using Seurat (version 5.4.0), and cytotoxic core scores were calculated using UCell (version 2.10.1). Data processing and visualization were performed using dplyr (version 1.2.0), tidyr, ggplot2 (version 4.0.2), and Matrix. Customized scripts were developed for pseudobulk construction, signature scoring, and paired cohort difference-in-differences (DiD) analyses.

### Ethics statement

This study used only previously published, publicly available, and de-identified datasets and did not involve new human subject recruitment or specimen collection. Therefore, this secondary analysis was exempt from additional institutional review board approval and informed consent requirements. The Xenium spatial transcriptomic analysis was performed on previously generated experimental data in accordance with the relevant institutional framework for animal experimentation.

## Results

### Cohort selection and analytical framework

**Fig. 1:**
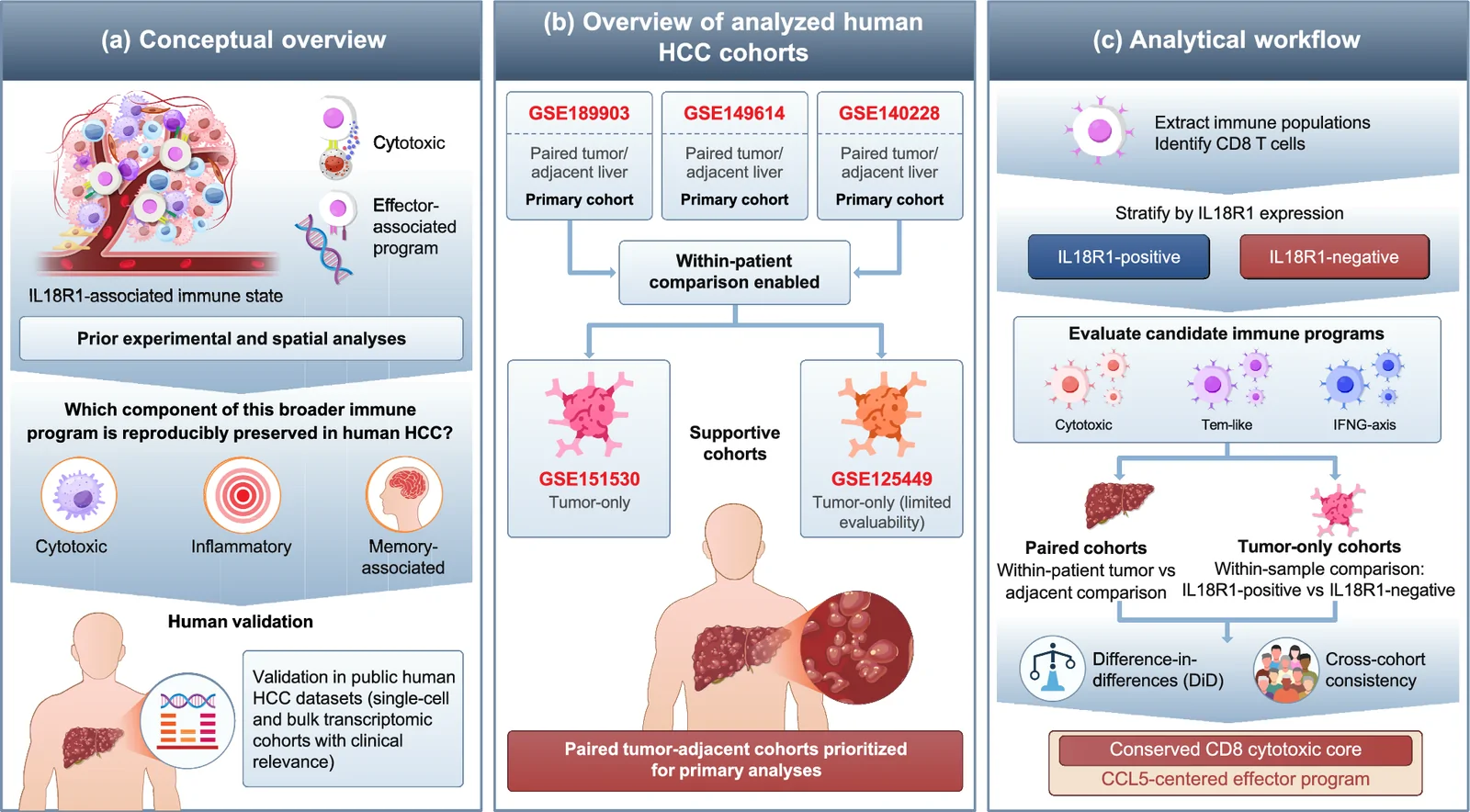
Study design and analytical framework for identifying a reproducible human correlate of an IL18R1-associated immune state in HCC, (a) Conceptual overview of the study. An IL18R1-associated immune state identified in previous experimental and spatial analyses was used as a reference framework for validation in public single-cell RNA-sequencing datasets of human HCC. (b) Overview of the analyzed human HCC cohorts. Datasets containing paired tumor and adjacent liver samples were prioritized for primary analyses, whereas tumor-only cohorts were used as supportive datasets. (c) Analytical workflow. CD8 T cells were identified, stratified according to IL18R1 expression, and compared using pseudobulk-based aggregation across cohorts. Paired datasets were analyzed using within-patient tumor-versus-adjacent contrasts, whereas tumor-only datasets were analyzed using within-sample IL18R1-positive versus IL18R1-negative comparisons. HCC, hepatocellular carcinoma; IL18R1, interleukin 18 receptor 1

To identify the conserved human correlate of an IL18R1-associated immune state in HCC, we analyzed multiple public single-cell RNA sequencing datasets using a unified analytical framework, prioritizing cohorts with paired tumor and adjacent liver samples for within-patient comparisons. Three paired cohorts (GSE189903, GSE149614, and GSE140228) were used for primary analyses, whereas two tumor-only cohorts (GSE151530 and GSE125449) served as supportive datasets to assess robustness and evaluability (Fig. 1a, b).

CD8 T cell populations were stratified according to IL18R1 expression status, and patient-level pseudobulk profiles were generated for downstream analyses (Fig. 1c). Tumor-specific enrichment was quantified using a DiD framework comparing IL18R1-associated contrasts between tumor and adjacent liver tissues. This approach enabled identification of immune-state components reproducibly associated with IL18R1 expression while minimizing interpatient variability.

### *Enrichment of a CCL5-linked cytotoxic program in IL18R1-positive CD8* + *T cells across paired HCC cohorts*

We first evaluated whether IL18R1-positive CD8 T cells were associated with cytotoxic transcriptional programs in paired human HCC cohorts. For each patient, differences in cytotoxic signature scores between IL18R1-positive and IL18R1-negative CD8 T cells were calculated and compared between tumor and adjacent liver tissues.

**Fig. 2:**
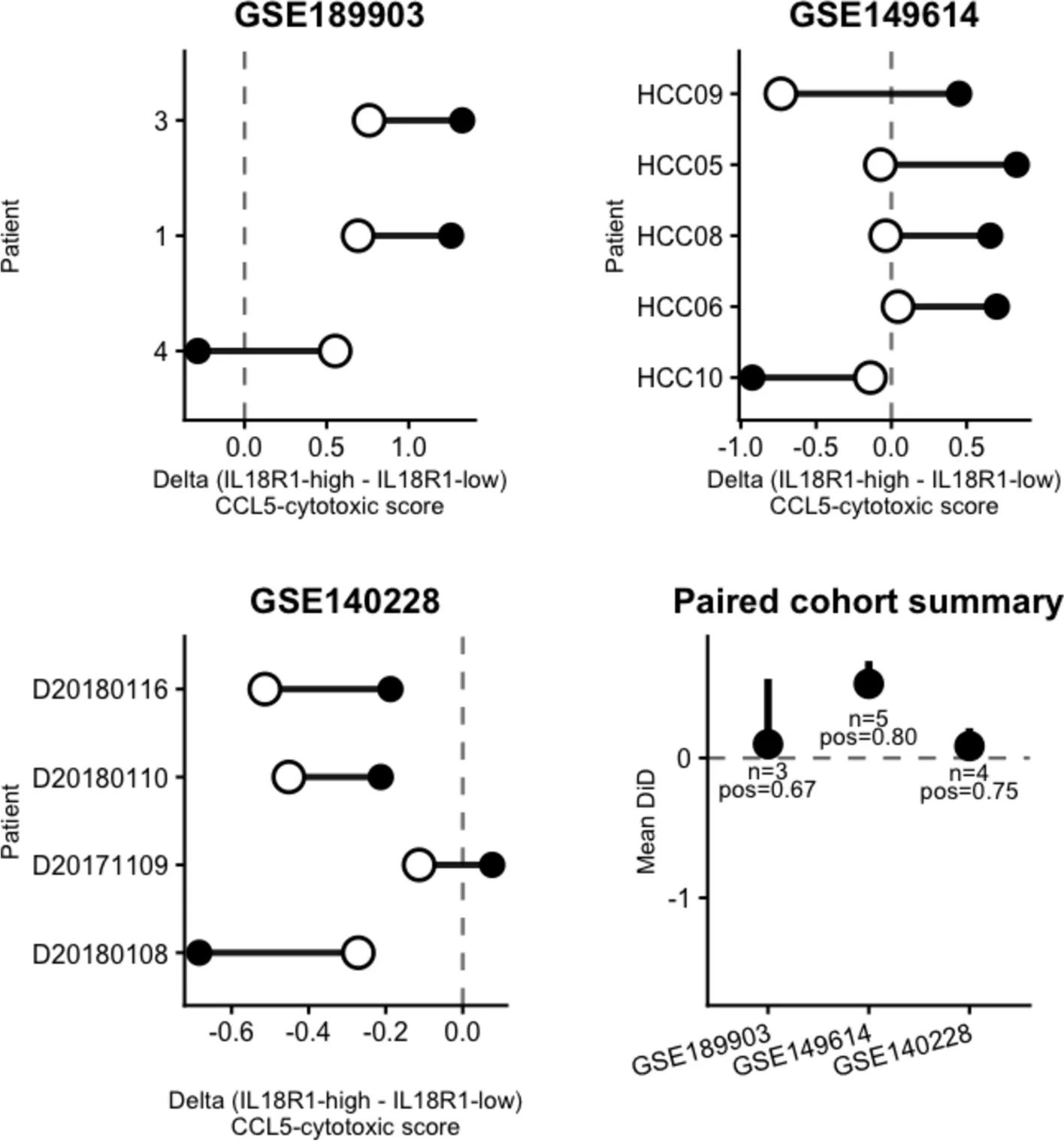
A conserved CCL5-centered CD8 cytotoxic module across paired human HCC cohorts, (a–c) Within-patient comparisons of IL18R1-positive versus IL18R1-negative CD8 + T cells across three independent paired HCC cohorts (GSE189903, GSE149614, and GSE140228). For each patient, differences in the CCL5-centered cytotoxic module scores were calculated separately for the tumor and adjacent liver tissues. Horizontal segments connect adjacent liver (open circles) and tumor (filled circles) values, illustrating tumor-specific shifts within individual patients. (d) Summary of tumor-specific enrichment across paired cohorts using a DiD framework. The mean DiD values are shown for each cohort, with labels indicating the number of patients analyzed and the fraction of patients showing positive enrichment. Across cohorts, IL18R1-positive CD8 + T cells exhibited consistent directional enrichment of a CCL5-centered cytotoxic transcriptional program, despite variability in effect size across datasets. CCL5, C–C motif chemokine ligand 5; DiD, difference-in-differences; HCC, hepatocellular carcinoma; IL18R1, interleukin 18 receptor 1

In GSE189903, most patients demonstrated positive enrichment of the CCL5-centered cytotoxic signature in IL18R1-positive CD8 T cells (Fig. 2a). Similar enrichment was observed in GSE149614 (Fig. 2b), whereas GSE140228 showed a comparable direction of effect with greater interpatient variability (Fig. 2c). Across all three paired cohorts, mean DiD values remained consistently positive (Fig. 2d), indicating tumor-specific enrichment of a CCL5-associated cytotoxic program within IL18R1-positive CD8 T cells.

These findings demonstrate a reproducible association between IL18R1 expression and a CCL5-centered cytotoxic transcriptional program across independent paired HCC cohorts.

### CD8-focused analytical modes improve recovery of the cytotoxic immune signal

**Fig. 3:**
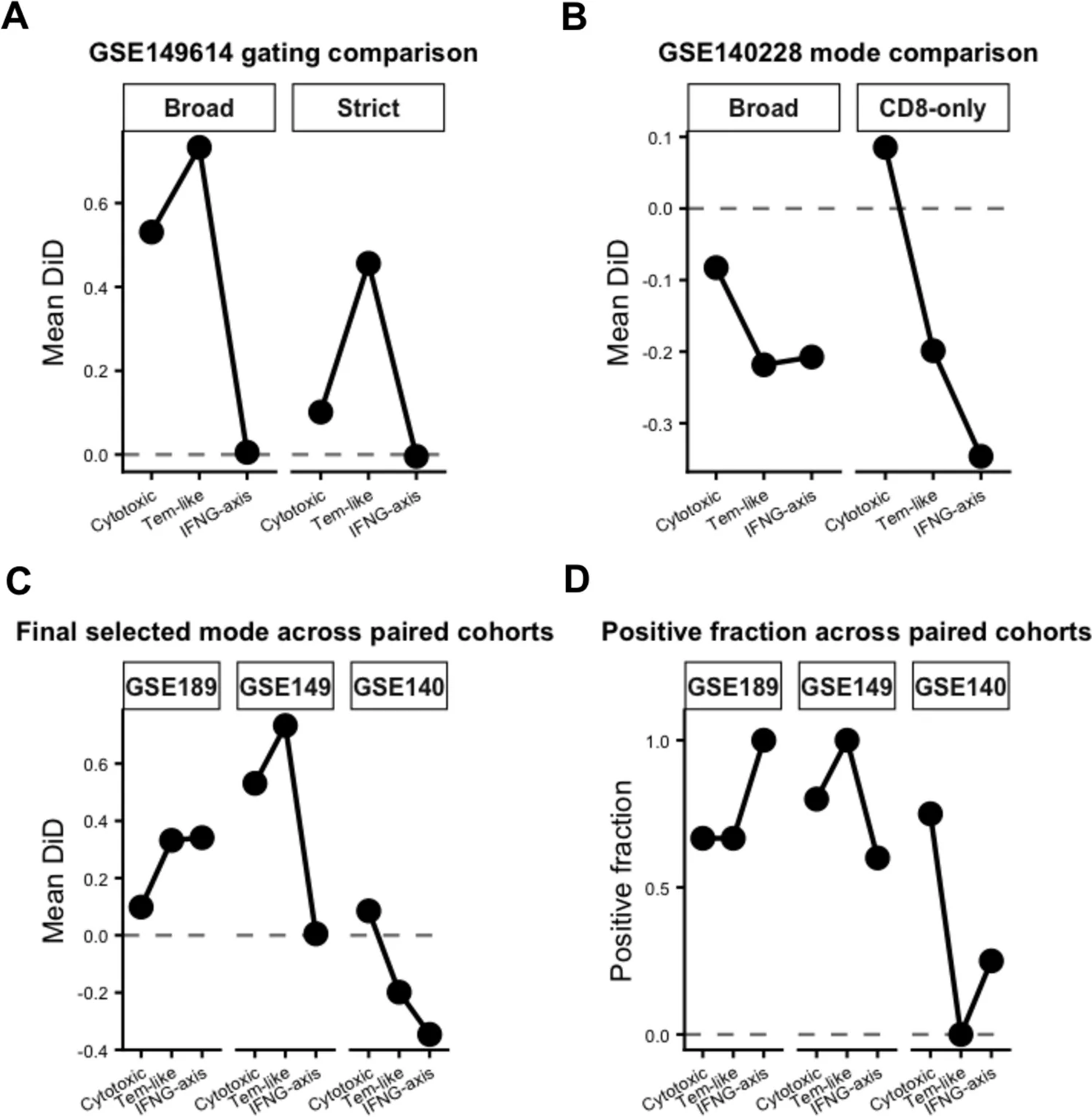
CD8-focused analytical strategies improve recovery of the conserved cytotoxic immune signal in HCC cohorts, (a) Gating comparison in GSE149614. Mean DiD values are shown for the cytotoxic, Tem-like, and IFNG-axis signatures under broad and strict gating strategies. (b) Compartment comparison in GSE140228. Mean DiD values are shown for the same three signatures under broad and CD8-only analytical modes. (c) Summary of the final selected analytical mode in each paired cohort (GSE189903, GSE149614, and GSE140228). Mean DiD values are shown for the cytotoxic, Tem-like, and IFNG-axis signatures. (d) Fraction of patients showing positive tumor-specific enrichment for each signature across paired cohorts. Across cohorts, the CCL5-centered cytotoxic signature showed the most reproducible positive enrichment, whereas broader IFNG-axis programs were less consistent. Restricting the analyses to CD8 compartments improved signal coherence relative to broader lymphoid analyses. Directionally consistent enrichment patterns were observed across both broad and strict CD8 T cell gating strategies, supporting the robustness of the identified cytotoxic program and arguing against a major contribution from NK-like populations. These findings support the robustness of the cytotoxic core, as shown in Fig. 2. CCL5, C–C motif chemokine ligand 5; DiD, difference-in-differences; HCC, hepatocellular carcinoma; IFNG, interferon-gamma; Tem, effector-memory T cell

To evaluate whether the observed immune program depended on cell-annotation strategy, we compared broad and strict CD8 T cell gating approaches in paired cohorts. In GSE149614, stricter CD8-focused analyses enhanced recovery of cytotoxic and effector-memory signals relative to broader lymphoid analyses (Fig. 3a). Similar findings were observed in GSE140228 (Fig. 3b).

Among the evaluated transcriptional programs, the CCL5-centered cytotoxic signature exhibited the most reproducible positive mean DiD values across cohorts (Fig. 3c) and the highest proportion of patients demonstrating positive enrichment (Fig. 3d). Directionally consistent enrichment patterns were observed across both broad and strict CD8 T cell gating strategies, indicating that the identified immune program was robust to cell-annotation stringency. These findings support a CD8-restricted cytotoxic module as the most stable component of the IL18R1-associated immune state and argue against the possibility that the observed signal was driven by the inclusion of NK-like populations.

### Tumor-only cohorts show greater heterogeneity in IL18R1-associated immune programs

**Fig. 4:**
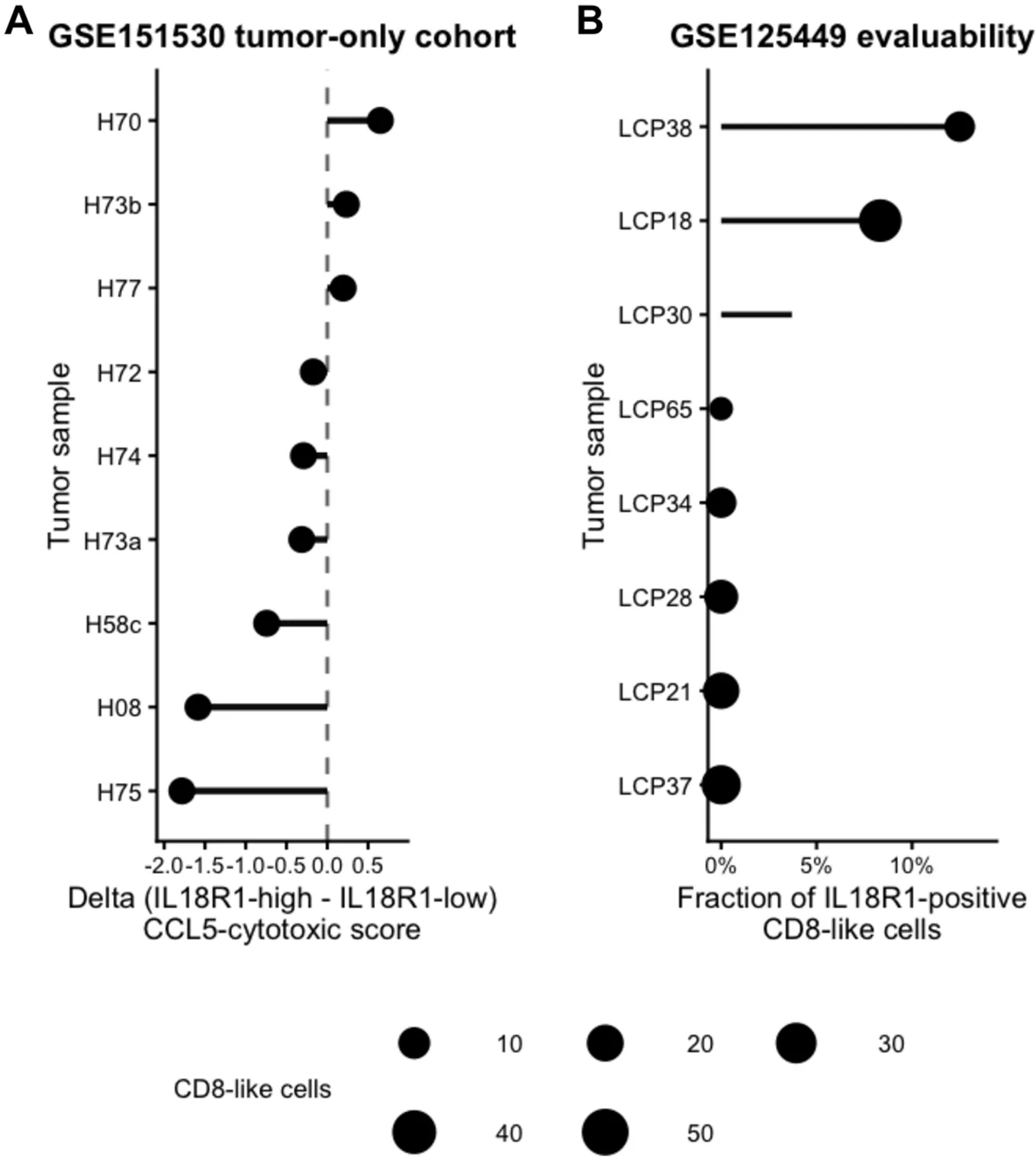
Tumor-only HCC cohorts reveal increased heterogeneity and limited evaluability of IL18R1-associated cytotoxic programs, (a) Tumor-only analysis of GSE151530 showing sample-level differences in CCL5-centered cytotoxic scores between IL18R1-positive and IL18R1-negative CD8 T cells. Each point represents a tumor sample, and horizontal segments indicate the magnitude and direction of the within-sample contrast. (b) Evaluability analysis of GSE125449 showing the fraction of IL18R1-positive CD8 T cells in each tumor sample. Point size indicates the number of CD8 T cells per sample. Compared to paired tumor-adjacent cohorts, tumor-only datasets showed greater heterogeneity in the direction and magnitude of the cytotoxic signal. In addition, sparse detection of IL18R1-positive CD8 T cells limited robust evaluation of GSE125449. These findings indicate that paired tumor–adjacent cohort designs provide a more reliable framework for resolving IL18R1-associated immune programs in human HCC. CCL5, C–C motif chemokine ligand 5; HCC, hepatocellular carcinoma; IL18R1, interleukin 18 receptor 1

To assess the reproducibility of the identified immune program in tumor-only datasets, we performed analogous analyses in GSE151530 and GSE125449. In GSE151530, IL18R1-positive CD8 T cells were detected but exhibited substantial variability in cytotoxic signature enrichment (Fig. 4a). Compared with paired cohorts, enrichment patterns were less consistent, resulting in a weaker overall support for the conserved cytotoxic program.

In GSE125449, sparse detection of IL18R1 transcripts substantially limited evaluability and precluded robust interpretation (Fig. 4b). Additional robustness analyses of alternative transcriptional signatures, gating strategies, and evaluability are provided in Supplementary Fig. S1. Collectively, these findings highlight the limitations of tumor-only datasets and support paired tumor-adjacent study designs for identifying conserved immune-state relationships in HCC.

### Clinical relevance of the CCL5-centered cytotoxic core in the TCGA-LIHC cohort

**Fig. 5:**
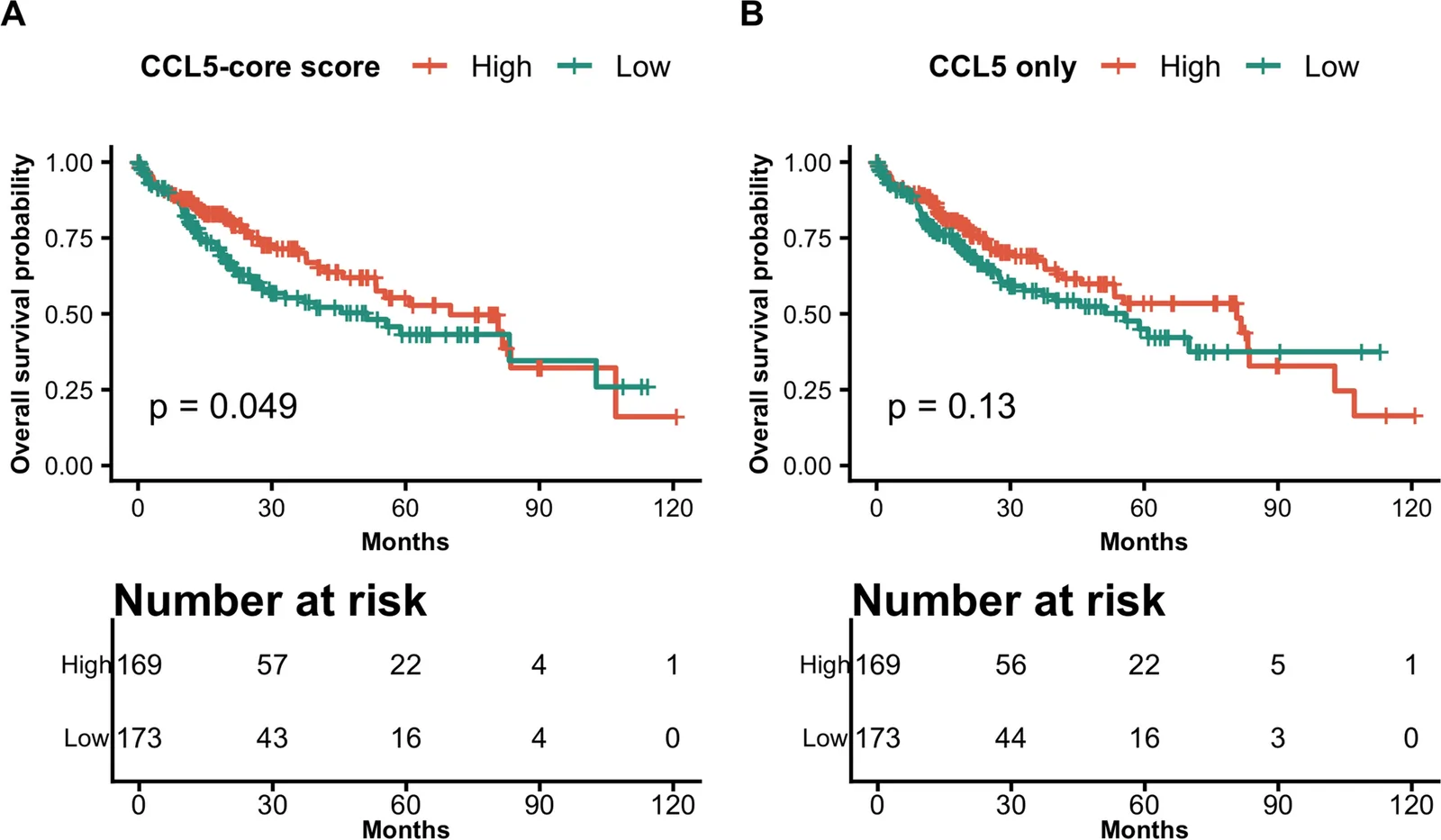
Clinical relevance of the CCL5-centered cytotoxic core in the TCGA-LIHC cohort, (a) Kaplan–Meier survival curves stratified by median CCL5-centered cytotoxic core score. (b) Kaplan–Meier survival curves stratified by CCL5 expression alone. The composite cytotoxic core, but not CCL5 alone, was associated with differences in overall survival in Kaplan–Meier analysis. Multivariable Cox regression analyses are provided in Supplementary Table S3. CCL5, C–C motif chemokine ligand 5; OS, overall survival; TCGA-LIHC, The Cancer Genome Atlas Liver Hepatocellular Carcinoma

To evaluate the clinical relevance of the conserved cytotoxic program, we analyzed bulk RNA sequencing data from the TCGA-LIHC cohort. Patients with high CCL5-centered cytotoxic core scores demonstrated significantly improved overall survival compared with those with low scores (log-rank *P* < 0.05; Fig. 5a). In contrast, CCL5 expression alone was not significantly associated with survival (Fig. 5b).

These findings suggest that coordinated cytotoxic immune programs may provide more clinically interpretable information than individual genes. In exploratory multivariable Cox regression analysis adjusting for age and AJCC stage, the association between the cytotoxic core score and overall survival was attenuated and did not reach statistical significance (HR 0.707, 95% CI 0.482–1.036; *P* = 0.076). Several advanced AJCC stage categories remained significantly associated with poor survival (Supplementary Table S3). Accordingly, the cytotoxic core is best interpreted as an immune stratification feature rather than an independent prognostic biomarker. Because IL18R1-specific CD8 T cell stratification is not feasible in bulk RNA-sequencing data, this analysis evaluated the clinical relevance of the conserved cytotoxic core as the human correlate of the IL18R1-associated state rather than the prognostic value of IL18R1 expression itself.

Because bulk transcriptomic data do not permit IL18R1-specific cellular stratification, the cytotoxic core score intentionally incorporated both cytotoxic effector genes and selected IFNG-inducible chemokines to capture the broader immune context associated with the conserved cytotoxic state rather than the reproducibility of individual signature components.

### Characterization and validation of the IL18R1-associated CD8 T cell state

To directly connect the murine spatial transcriptomic dataset with the human IL18R1-associated immune program, Xenium CD8 T cells were stratified according to IL18R1 expression status. IL18R1-positive cells were broadly distributed across CD8 T cell subsets and spatial compartments, indicating that they represent a spatially resolvable population rather than a rare or isolated subset (Supplementary Fig. S2). Within tumor regions, IL18R1-high CD8 T cells exhibited significantly increased expression of Ccl5 and Cxcr3 compared with IL18R1-low CD8 T cells (Supplementary Fig. S3A–C). Similar enrichment of Ccl5 expression remained evident when analyses were restricted to treatment-associated tumor regions (Supplementary Fig. S3D).

Moreover, the proportion of IL18R1-high CD8 T cells was significantly greater in treated tumors than in control tumors (Supplementary Fig. S3E; Fisher’s exact test *P* = 0.0119). Importantly, these findings remained consistent when applying an alternative quartile-based IL18R1 stratification strategy to IL18R1-positive CD8 T cells, indicating that the identified immune program was not dependent on the primary binary IL18R1 threshold.

These IL18R1-associated activation features and components of the cytotoxic program remained detectable after adjustment for transcript detection depth in nFeature_RNA-adjusted logistic regression analyses (Supplementary Fig. S4 and Supplementary Table S4). Together, these analyses provide orthogonal spatial evidence linking the murine Xenium observations to the IL18R1-associated CD8 T cell state identified in human HCC cohorts.

### Spatial validation of the CD8 cytotoxic core in a liver tumor model

Having established that IL18R1-high CD8 T cells are specifically enriched for the cytotoxic program in this dataset (Supplementary Fig. S3), we next evaluated whether the conserved cytotoxic core itself was biologically and spatially observable by analyzing the same Xenium spatial transcriptomic dataset from a murine liver tumor model.

**Fig. 6:**
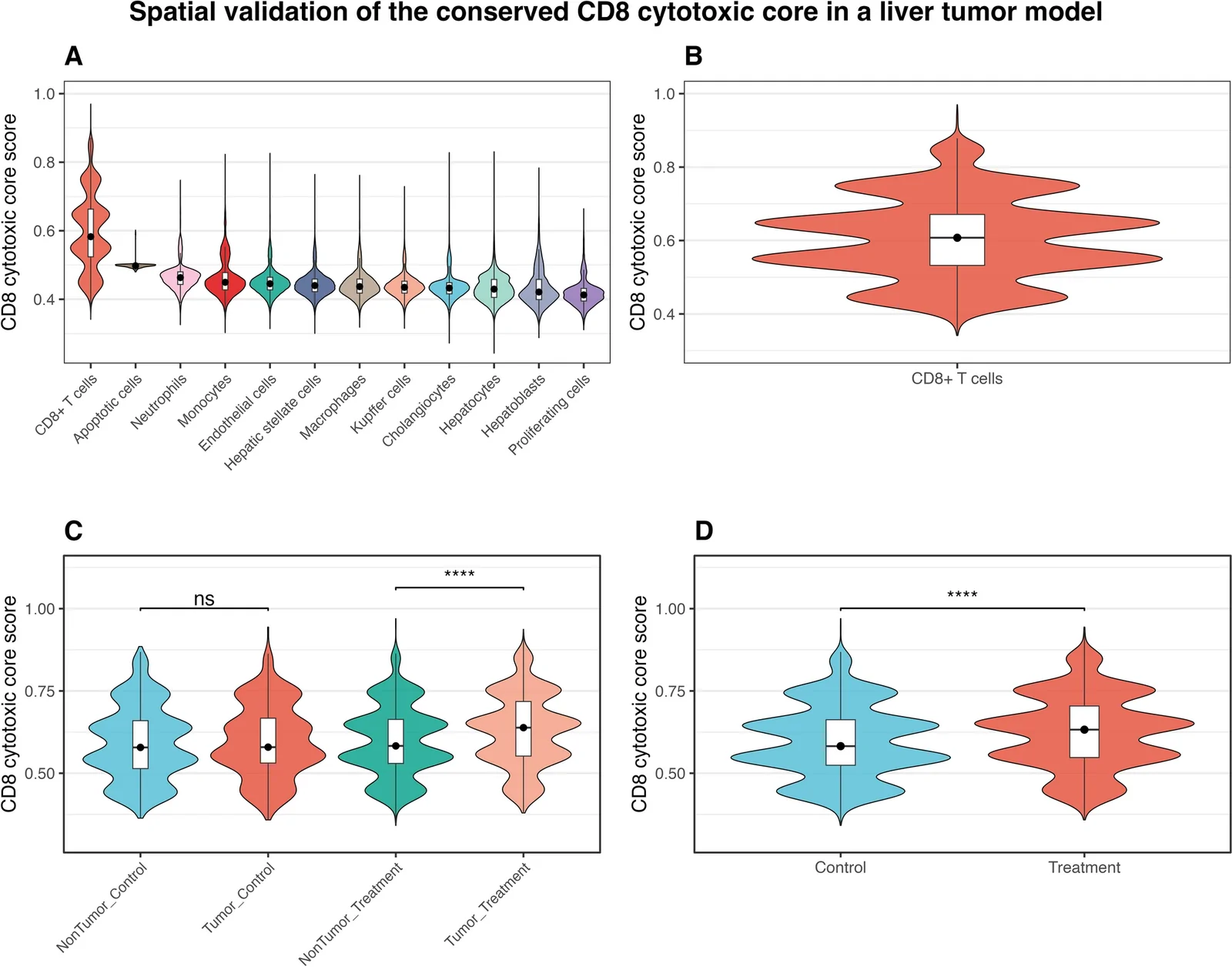
Spatial validation of the conserved CD8 cytotoxic core in a liver tumor model, (a) Distribution of CD8 cytotoxic core scores across major cell types in the Xenium spatial transcriptomic dataset. CD8 + T cells exhibited the highest cytotoxic core scores, indicating preferential enrichment of this program within the CD8 compartment, although lower-level signals were also detectable in other cell populations. (b) Distribution of CD8 cytotoxic core scores within CD8 + T cells. The scores showed a broad and continuous distribution without a clearly separable discrete high-score subpopulation, indicating heterogeneity in cytotoxic potential within the CD8 T cell compartment. (c) Comparison of CD8 cytotoxic core scores across spatial regions within CD8 + T cells. No significant difference was observed between non-tumor and tumor regions in the control group (ns), whereas a significant increase in tumor regions compared to non-tumor tissues was observed under treatment conditions (****), indicating context-dependent spatial modulation of this immune program. (d) Comparison of CD8 cytotoxic core scores between experimental groups within CD8 + T cells. A statistically significant increase in cytotoxic core scores was observed in the treatment group compared with the control group (****), supporting the biological relevance and dynamic nature of this conserved immune-state unit. These analyses were not intended to evaluate treatment efficacy. Cytotoxic core scores were calculated at the single-cell level using a predefined gene signature (*CCL5, PRF1, GZMB, NKG7, GNLY, GZMK, XCL1, XCL2,* and *CXCR3*) adapted to the Xenium panel and summarized using violin plots with overlaid boxplots. Statistical comparisons were performed using the Wilcoxon rank-sum test. ns, not significant; ****, *p* < 0.0001

The CD8 cytotoxic core score was the highest in CD8 T cells, indicating preferential enrichment within this compartment (Fig. 6a). Nevertheless, detectable scores were also observed in other immune and non-immune populations, suggesting a CD8-biased rather than CD8-exclusive immune state. Within CD8 T cells, cytotoxic core scores exhibited substantial heterogeneity without evidence of a discrete high-score subpopulation (Fig. 6b).

Spatial analyses revealed heterogeneous distributions of the cytotoxic program. No significant difference was observed between tumor and non-tumor regions in control samples, whereas tumor regions from treated animals demonstrated increased cytotoxic core scores (Fig. 6c). A significant increase in cytotoxic core scores under treatment conditions further supported the biological relevance and modifiability of this immune state (Fig. 6d), although these analyses were not intended to evaluate treatment efficacy.

Representative spatial maps additionally demonstrated localization of Cd8a-positive cells together with Ccl5-high and Gzmb-high CD8 T cell populations within tumor regions (Supplementary Fig. S5), providing visual support for the spatial localization of the conserved cytotoxic core identified across human HCC cohorts.

## Discussion

This study identified a conserved CD8 cytotoxic core as the dominant human correlate of an IL18R1-associated immune state in HCC. Across independent paired HCC cohorts, a compact cytotoxic module enriched for CCL5 and effector-associated genes emerged as the most reproducibly conserved component of the IL18R1-associated program, whereas broader IFNG-associated inflammatory signatures demonstrated greater variability. These findings suggest that reproducible immune states in human tumors may be better represented by compact transcriptional modules than by broadly defined inflammatory programs.

Our findings help identify biologically interpretable immune-state units that remain reproducible across heterogeneous human datasets. HCC is characterized by substantial molecular and immune heterogeneity, and previous studies have identified multiple cytotoxic, exhausted, memory-like, and regulatory T cell states within the tumor microenvironment [6–8]. However, many immune signatures vary considerably across cohorts and analytical frameworks. In contrast, the CCL5-centered cytotoxic module identified in the present study demonstrated consistent enrichment across independent paired datasets and remained evident under multiple analytical strategies, suggesting that it represents a particularly stable component of the IL18R1-associated immune landscape.

The biological plausibility of this association is supported by established functions of IL-18 signaling. IL-18 promotes type 1 immune responses and enhances cytotoxic lymphocyte activity through IL18R1–IL18RAP receptor complex-mediated signaling [9, 10]. Nevertheless, the present study was not designed to establish a direct causal regulatory relationship between IL-18 signaling and the observed cytotoxic transcriptional program. Rather, our findings define a reproducible immune-state association that is conserved across independent human cohorts and supported by spatial transcriptomic analyses. Accordingly, the identified cytotoxic module should be interpreted as the dominant conserved correlate of an IL18R1-associated immune state rather than as definitive evidence of a direct IL-18-driven transcriptional program.

Importantly, the observed enrichment patterns remained directionally consistent when analyses were restricted to more stringently defined CD8 T cell compartments. These findings indicate that the identified cytotoxic program is robust to the cell-annotation strategy and argue against the possibility that the observed associations were driven by the inclusion of NK-like populations. Together with the reproducibility observed across independent cohorts, this supports the biological specificity of the identified CD8 cytotoxic module.

Our analyses also revealed a coordinated association between IL18R1 expression and a CCL5-centered cytotoxic transcriptional program. Because the study relied primarily on observational transcriptomic datasets, these findings should be interpreted as evidence of a conserved immune-state relationship rather than proof of a direct signaling hierarchy. Current models of T cell differentiation indicate that activated, memory-like, exhausted, and cytotoxic states often overlap and share substantial transcriptional features [11, 15, 16]. Within this context, IL18R1 expression appears to identify a subset of CD8 T cells preferentially enriched for cytotoxic effector-associated genes. Consistent with this interpretation, IL18R1-positive CD8 T cells in the Xenium dataset co-expressed activation and exhaustion markers such as programmed cell death 1 (Pdcd1), lymphocyte-activation gene 3 (Lag3), and thymocyte selection-associated high mobility group box (Tox) alongside effector genes, a profile more consistent with a progenitor-exhausted or transitional state in which effector gene expression is retained, rather than a terminally exhausted population that has lost cytotoxic effector function. Whether this relationship reflects direct IL-18-mediated regulation or a broader activated CD8 T cell state remains to be determined.

The recurrent identification of CCL5 within this immune program is biologically noteworthy. Chemokine networks play central roles in orchestrating antitumor immunity by regulating the recruitment, positioning, and retention of effector lymphocytes within tumor tissues. CCL5 has been implicated in T cell trafficking and cytotoxic immune activity and may cooperate with IFNγ-inducible chemokines, particularly CXCL9 and CXCL10, to promote the accumulation of effector T cells within tumors [13, 17]. The consistent enrichment of a CCL5-centered cytotoxic module across independent cohorts, therefore, suggests that this transcriptional program captures an important aspect of tumor-associated cytotoxic immune activity.

A major strength of this study is the use of paired tumor-adjacent cohorts to define immune-state relationships. Compared with tumor-only datasets, paired designs permit within-patient comparisons and reduce confounding by baseline interpatient immune variability. Consistent enrichment of the cytotoxic program across multiple paired cohorts contrasted with the greater heterogeneity observed in tumor-only datasets, emphasizing the importance of study design when evaluating immune states in heterogeneous tumors.

Spatial transcriptomic analyses provided additional biological support for the identified immune-state unit. Xenium analyses demonstrated that IL18R1-high tumor-associated CD8 T cells were enriched for Ccl5 and Cxcr3 expression and were more frequent in treatment-associated tumor regions. These findings support an association between the murine spatial observations and the IL18R1-associated CD8 T cell state identified in human HCC cohorts. Moreover, cytotoxic core scores were preferentially enriched within CD8 T cells and remained spatially observable within tumor regions. Representative spatial maps further demonstrated localization of Cd8a-positive cells together with Ccl5-high and Gzmb-high CD8 T cell subsets, providing visual support for the biological observability of the conserved cytotoxic program.

Although a modest increase in cytotoxic core scores was observed under treatment conditions in the Xenium dataset, these analyses were not intended to establish treatment efficacy. Rather, they demonstrate that the conserved immune-state unit identified in human datasets can be dynamically modulated within an experimental tumor context. Similarly, the TCGA-LIHC analyses suggest that coordinated cytotoxic immune programs may provide more clinically interpretable information than individual genes. However, because the association between the cytotoxic core score and overall survival was attenuated after adjustment for clinical covariates, the identified cytotoxic module should not be interpreted as an independent prognostic biomarker. Instead, it should be viewed as a reproducible immune-state feature that captures biologically meaningful variation in tumor-associated CD8 T cell activity.

This study has several limitations. First, human analyses relied on publicly available datasets generated using different experimental protocols, which may have introduced technical variability. Second, spatial validation was limited to a single murine Xenium dataset and was not replicated in independent human spatial transcriptomic cohorts. Therefore, the spatial findings should be interpreted as biological validation rather than proof of spatial conservation in human HCC. Third, sparse detection of IL18R1 transcripts in several datasets limited evaluability and necessitated a primary binary IL18R1-positive versus IL18R1-negative classification strategy. Although additional analyses using alternative stratification approaches and nFeature_RNA-adjusted models supported the robustness of the findings, residual effects of transcript detection variability cannot be completely excluded [18]. Relatedly, IL18R1 status was defined at the transcript level, and orthogonal protein-level confirmation, such as multiplex immunohistochemistry or immunofluorescence, was not performed. Nevertheless, analysis of the Xenium spatial transcriptomic dataset, an in situ single-molecule platform that does not require tissue dissociation, confirmed that IL18R1-expressing CD8 T cells constitute a spatially resolvable population distributed across CD8 T cell subsets (Supplementary Fig. S2) and enriched for the cytotoxic program (Supplementary Fig. S3), providing single-cell, in situ support for the existence of this population independent of dissociation-based dropout. Finally, the study was not designed to establish causality. Functional perturbation experiments, including IL-18 stimulation, IL18R1 blockade, or genetic manipulation of the IL18R1 pathway, will be required to determine whether the observed CCL5-centered cytotoxic program is directly regulated by IL-18 signaling.

Across independent human HCC cohorts, the most reproducible component of an IL18R1-associated immune state was a compact CD8 cytotoxic module enriched for CCL5 and effector-associated genes. This CCL5-centered cytotoxic core demonstrated greater cross-cohort reproducibility than broader inflammatory programs and remained biologically observable in spatial transcriptomic analyses. More broadly, our findings provide a framework for decomposing complex tumor immune states into minimal, reproducible immune-state units that can be validated across independent datasets and spatial contexts. This framework may facilitate the identification of biologically interpretable immune programs and improve immune stratification strategies in HCC and other heterogeneous tumor types.

## Supplementary Information

Below is the link to the electronic supplementary material.

Supplementary file1 (PNG 1114 KB)

Supplementary file2 (PNG 1348 KB)

Supplementary file3 (PNG 1352 KB)

Supplementary file4 (PNG 1380 KB)

Supplementary file5 (PNG 1264 KB)

Supplementary file6 (DOCX 32 KB)

## Data Availability

All datasets analyzed in this study are publicly available. Single-cell RNA sequencing datasets were obtained from the Gene Expression Omnibus (GEO), including GSE189903, GSE149614, GSE140228, GSE151530, and GSE125449. Bulk transcriptomic and corresponding clinical data for TCGA-LIHC were downloaded through TCGAbiolinks. The processed matrices and derived analytical tables used in this study are available from the corresponding author upon request.

## Abbreviations

B2M: Beta-2 microglobulin
CCL5: C–C motif chemokine ligand 5
CPM: Counts per million
CXCL: Chemokine C-X-C motif ligand
CXCR: CXC chemokine receptor
DiD: Difference-in-differences
GBP: Guanylate-binding protein
GEO: Gene Expression Omnibus
GNLY: Granulysin
GZM: Granzyme
HCC: Hepatocellular carcinoma
HLA-DRA: HLA class II histocompatibility antigen, DR alpha
IFNG: Interferon-gamma
IL-18: Interleukin-18
IL18RAP: IL-18 receptor accessory protein
IRF1: Interferon regulatory factor 1
MyD88: Myeloid differentiation primary response 88
NKG7: Natural killer cell granule protein 7
PD-L1: Programmed death-ligand 1
PRF1: Perforin 1
STAT4: Signal transducer and activator of transcription 4
XCL: XC motif chemokine ligand

## Code availability

The datasets analyzed in this study are publicly available from the Gene Expression Omnibus (GEO) under accession numbers GSE189903, GSE149614, GSE140228, GSE151530, and GSE125449, and from The Cancer Genome Atlas Liver Hepatocellular Carcinoma (TCGA-LIHC) cohort. The Xenium spatial transcriptomics dataset generated in this study is available from the corresponding author upon reasonable request.
