# Supplementary material for: Conserved minimal CD8 cytotoxic immune-state unit in an IL18R1-associated immune program in hepatocellular carcinoma
Source: Cancer Immunol Immunother. 2026 Jul 6;75(10):238. doi: 10.1007/s00262-026-04487-9 (PMC13620086; doi:10.1007/s00262-026-04487-9)
Supplement: Supplementary file 1 [file 262_2026_4487_MOESM6_ESM.docx]

**Conserved minimal CD8 cytotoxic immune-state unit in an IL18R1-associated immune program in hepatocellular carcinoma**

**Supplementary Figure S1.** Robustness and evaluability analyses of IL18R1-associated cytotoxic programs in tumor-only cohorts

(A) Evaluation of secondary transcriptional signatures in the tumor-only cohort GSE151530. While the Tem-like signature showed a general trend toward positive enrichment, the IFNG-axis signature exhibited substantial variability and lacked consistent directional enrichment, indicating reduced robustness of broader inflammatory programs in tumor-only settings.

(B) Patient-level differences in CCL5-centered cytotoxic signature scores (IL18R1-positive vs. IL18R1-negative) in GSE125449. Although directional differences could be detected in some patients,

the sparse representation of IL18R1-positive CD8-like cells substantially limited evaluability and robust interpretation.

(C) Comparison of cytotoxic, Tem-like, and IFNG-axis signatures in GSE125449. Among the evaluated programs, the cytotoxic signature demonstrated the most consistent directional trend, whereas the IFNG-axis signature showed substantial variability.

(D) Sample-level comparison of CCL5-centered cytotoxic signature scores between IL18R1-negative and IL18R1-positive CD8-like cells in GSE125449. Individual samples showed heterogeneous but detectable shifts in cytotoxic activity associated with IL18R1 expression.

Collectively, these analyses demonstrate that tumor-only cohorts exhibit increased heterogeneity and reduced evaluability compared to paired datasets, while the CCL5-centered cytotoxic program remains the most consistently detectable component across analytical conditions

Abbreviations: CCLl5: C-C motif chemokine ligand 5; IFNG: interferon gamma; IL18R1, interleukin-18 receptor 1

**Supplementary Figure S2.** Characterization of IL18R1-positive CD8 T cells in the Xenium spatial transcriptomic dataset.

(A) UMAP visualization of IL18R1 expression across CD8 T cells. Cells are colored according to normalized IL18R1 expression levels.

(B) Proportion of IL18R1-positive CD8 T cells across spatial regions, including non-tumor control, non-tumor treatment, tumor control, and tumor treatment compartments.

(C) Absolute numbers of IL18R1-positive and IL18R1-negative CD8 T cells within each spatial region.

(D) Frequency of IL18R1-positive cells across CD8 T-cell subsets. IL18R1-positive cells were detected in all CD8 T-cell compartments, with the highest proportion observed in the CD8 Tex population.

These analyses demonstrate that IL18R1-positive CD8 T cells are broadly distributed across CD8 T-cell subsets and spatial compartments and are not restricted to a rare or isolated cellular population, supporting their suitability for downstream characterization of IL18R1-associated cytotoxic programs.

Abbreviations: CD8 Tex: exhausted CD8 T cells; IL18R1, interleukin-18 receptor 1; UMAP: Uniform Manifold Approximation and Projection

**Supplementary Figure S3.** IL18R1-high CD8 T cells are associated with a cytotoxic transcriptional program in tumor regions as revealed by Xenium spatial transcriptomics.

(A–C) Expression of Ccl5, Gzmb, and Cxcr3 in tumor-region, and CD8 T cells stratified according to IL18R1 expression level. IL18R1-low and IL18R1-high populations were defined as the lower and upper quartiles of IL18R1-positive CD8 T cells, respectively.

(D) Ccl5 expression in tumor-region, and CD8 T cells restricted to treatment-associated tumors. Enrichment of Ccl5 expression remained evident within the IL18R1-high compartment.

(E) Frequency of IL18R1-high CD8 T cells among tumor-region IL18R1-positive CD8 T cells. Treatment tumors contained a significantly higher proportion of IL18R1-high CD8 T cells than did control tumors (Fisher’s exact test, P = 0.0119).

These analyses provide orthogonal spatial evidence linking the murine Xenium observations to the IL18R1-associated CD8 T-cell state identified in human HCC cohorts and demonstrate that the IL18R1-associated immune program is enriched within tumor-associated CD8 T cells in vivo.

The observed enrichment patterns remained evident using an alternative quartile-based IL18R1 stratification strategy among IL18R1-positive CD8 T cells and therefore were not dependent on the primary binary IL18R1-positive versus IL18R1-negative classification.

Abbreviations: ccl5: C-C motif chemokine ligand 5; Cxcr3: CXC chemokine receptor 3; Gzmb: granzyme B; IL18R1, interleukin-18 receptor 1

**Supplementary Figure S4.** Detection-depth sensitivity analysis of IL18R1-associated CD8 T-cell states.

(A) Distribution of nFeature_RNA in IL18R1-negative and IL18R1-positive CD8 T cells.

(B) Distribution of nCount_RNA in IL18R1-negative and IL18R1-positive CD8 T cells.

(C) Relationship between nFeature_RNA and Ccl5 expression in CD8 T cells. Spearman’s correlation coefficient (rho) is shown.

(D) Relationship between nFeature_RNA and Gzmb expression in CD8 T cells. Spearman’s correlation coefficient (rho) is shown.

(E) nFeature_RNA-adjusted differential expression analysis comparing IL18R1-positive and IL18R1-negative CD8 T cells using logistic regression. Positive avg_log2FC values indicate higher expression in IL18R1-positive cells, whereas negative values indicate higher expression in IL18R1-negative cells.

These analyses were performed to evaluate whether the IL18R1-associated transcriptional program could be explained by transcript detection depth or RNA-content bias. Multiple activation- and cytotoxicity-associated genes remained significantly associated with IL18R1-positive cells after adjustment for nFeature_RNA. Consistent with an antigen-experienced effector state, IL18R1-positive CD8 T cells also showed higher expression of activation and exhaustion markers, including Ifng, Cxcr3, Prf1, Pdcd1, Lag3, and Tox, whereas Ccl5 did not reach significance in this binary, whole-tumor differential expression analysis. In contrast, the tumor-restricted quartile-based stratification shown in Supplementary Fig. S3 demonstrated significant Ccl5 enrichment in IL18R1-high cells, suggesting that the binary result likely reflects sparse and dropout-prone transcript detection rather than a true absence of association.

Abbreviations: ccl5: C-C motif chemokine ligand 5; Cxcr3: CXC chemokine receptor 3; Gzmb: granzyme B; Ifng, interferon gamma; IL18R1, interleukin-18 receptor 1; Lag3: lymphocyte-activation gene 3; Pdcd1: programmed cell death 1; Prf1: perforin 1; Tox: thymocyte selection-associated high mobility group box.

**Supplementary Figure S5.** Representative spatial localization of Cd8a+ cells and cytotoxic-program-associated CD8+ T-cell populations in Xenium spatial transcriptomic data.

(A) Spatial distribution of Cd8a-positive cells in tumor regions from control and IL-2 + IL-18 + anti-PD-L1-treated mice.

(B) Spatial localization of Ccl5-high CD8+ T cells, defined as the top 10% of CD8+ T cells ranked by Ccl5 expression.

(C) Spatial localization of Gzmb-high CD8+ T cells, defined as the top 10% of CD8+ T cells ranked by Gzmb expression.

Gray dots indicate all cells within the annotated tumor region. Colored dots indicate the highlighted cell population, with color intensity corresponding to normalized transcript expression levels. These representative spatial maps provide visual support for the spatial observability of the conserved cytotoxic CD8 program within tumor regions.

Abbreviations: ccl5: C-C motif chemokine ligand 5; Gzmb: granzyme B; IL18R1, interleukin-18 receptor 1

**Supplementary Table S1. Characteristics of the cohorts included in the present study**

| Cohort | Platform | Study design | Original cohort size | Samples included in analysis | Main analytical use |
| --- | --- | --- | --- | --- | --- |
| GSE189903 | Single-cell RNA sequencing | Paired tumor–adjacent liver | 4 HCC patients | 3 patients | Primary paired cohort |
| GSE149614 | Single-cell RNA sequencing | Paired tumor–adjacent liver | 10 HCC patients | 5 patients | Primary paired cohort |
| GSE140228 | Single-cell RNA sequencing | Paired tumor–adjacent liver | 10 HCC patients | 4 patients | Primary paired cohort |
| GSE151530 | Single-cell RNA sequencing | Tumor-only | 25 HCC patients | 9 evaluable tumor samples | Tumor-only validation cohort |
| GSE125449 | Single-cell RNA sequencing | Tumor-only | 9 HCC patients | 7 evaluable patients | Exploratory tumor-only cohort |
| TCGA-LIHC | Bulk RNA sequencing | Tumor-only | 363 patients | 342 patients | Survival analysis |
| Xenium (internal dataset) | Spatial transcriptomics | Paired mouse liver tumor model | 2 mice | 2 mice | Spatial validation |

**Abbreviations:** HCC, hepatocellular carcinoma; LIHC, liver hepatocellular carcinoma.

**Footnote:** Original cohort sizes were obtained from the corresponding source publications or Gene Expression Omnibus records. Samples included in analysis represent patients or samples that passed dataset-specific quality-control criteria and were evaluable for IL18R1-based comparisons. For paired analyses, patients were required to contain both IL18R1-high and IL18R1-low CD8 T-cell populations with sufficient cell numbers for pseudobulk comparison. The Xenium dataset consisted of two mice (one vehicle-treated control and one IL-2 + IL-18 + anti-PD-L1–treated mouse), each containing paired tumor and non-tumor regions of interest.

Supplementary Table S2. Definition of gene signatures used in this study

| Signature name | Genes included | Main analytical use |
| --- | --- | --- |
| CCL5-linked cytotoxic signature (primary) | *CCL5, PRF1, GZMB, GZMH, GNLY, NKG7, XCL1, XCL2, HLA-DRA,* and *B2M* | Primary paired-cohort analyses evaluating tumor-specific IL18R1-associated cytotoxic enrichment (Figs. 2–4) |
| Effector-memory (Tem-like) signature | *CCL5, NKG7, GNLY, PRF1, GZMB, GZMK, CXCR3,* and *IFNG* | Alternative signature robustness analyses and gating comparisons (Fig. 3) |
| IFNG-associated signature | *CCL5, IFNG, STAT1, IRF1, CXCL9, CXCL10, CXCL11, GBP1, GBP2,* and *B2M* | Alternative signature robustness analyses and gating comparisons (Fig. 3) |
| CCL5-centered cytotoxic core score | *CCL5, GZMA, GZMB, PRF1, NKG7, IFNG, CXCL9,* and *CXCL10* | TCGA-LIHC bulk RNA-seq survival analyses (Fig. 5) |
| CD8 cytotoxic core score (spatial) | *CCL5, PRF1, GZMB, NKG7, GNLY, GZMK, XCL1, XCL2,* and *CXCR3* | Xenium spatial transcriptomic analyses of murine liver tumors (Fig. 6) |

Abbreviations: IFNG, interferon-γ; Tem, effector-memory T cell.

Gene signatures were predefined prior to comparative analyses. The CCL5-linked cytotoxic signature was used as the primary signature throughout the paired-cohort analyses because it demonstrated the most consistent tumor-specific enrichment across independent human datasets. Effector-memory and IFNG-associated signatures were used as secondary robustness analyses. The CCL5-centered cytotoxic core score was applied to TCGA-LIHC bulk RNA-seq data for survival analyses, whereas the CD8 cytotoxic core score was used for spatial validation in the Xenium dataset.

| **Supplementary Table S3. Multivariable Cox regression analysis for overall survival in the TCGA-LIHC cohort.** | | | |
| --- | --- | --- | --- |
| Variable | HR | 95% CI | P value |
| CCL5-core high vs. low | 0.707 | 0.482–1.036 | 0.076 |
| Age (per year) | 1.014 | 0.999–1.030 | 0.073 |
| AJCC Stage II vs. I | 1.49 | 0.905–2.451 | 0.117 |
| AJCC Stage III vs. I | 1.337 | 0.312–5.731 | 0.696 |
| AJCC Stage IIIA vs. I | 2.637 | 1.639–4.241 | <0.001 |
| AJCC Stage IIIB vs. I | 2.724 | 0.967–7.672 | 0.058 |
| AJCC Stage IIIC vs. I | 3.099 | 1.097–8.759 | 0.033 |
| AJCC Stage IV vs. I | 7.696 | 1.833–32.311 | 0.005 |
| AJCC Stage IVA vs. I | NE | NE | 0.997 |
| AJCC Stage IVB vs. I | 4.143 | 0.995–17.247 | 0.051 |
| NE : Not Estimable |  |  |  |
|  |  |  |  |
| Abbreviations: AJCC, American Joint Committee on Cancer; CCL5, C-C motif chemokine ligand 5; HR, hazard ratio; CI, confidence interval; NE, not estimable; TCGA-LIHC, The Cancer Genome Atlas Liver Hepatocellular Carcinoma  AJCC stage I was used as the reference category.  AJCC stages were analyzed according to the original TCGA clinical annotations. | | | |

| **Supplementary Table S4. Differential expression analysis between IL18R1-positive and IL18R1-negative CD8 T cells in the Xenium dataset adjusted for nFeature_RNA.** | | | | | |
| --- | --- | --- | --- | --- | --- |
| Gene | P value | Average log2FC | Pct. IL18R1-positive | Pct. IL18R1-negative | Adjusted P value |
| *Tox* | 8.20747E-31 | 1.254477539 | 0.403 | 0.369 | 3.93138E-28 |
| *Cd8a* | 1.76245E-08 | 1.207287871 | 0.599 | 0.615 | 8.44214E-06 |
| *Cxcr3* | 2.11036E-07 | 1.05894456 | 0.45 | 0.477 | 0.000101086 |
| *Pdcd1* | 4.1669E-25 | 1.036400344 | 0.305 | 0.284 | 1.99594E-22 |
| *Lag3* | 6.09025E-20 | 0.935204203 | 0.226 | 0.24 | 2.91723E-17 |
| *Ifng* | 4.41429E-09 | 0.87861608 | 0.157 | 0.148 | 2.11445E-06 |
| *Prf1* | 1.22773E-06 | 0.666704523 | 0.235 | 0.286 | 0.000588083 |
| *Ccl5* | 0.295006827 | 0.643095435 | 0.683 | 0.807 | 1 |
| *Gzmb* | 2.65316E-08 | −0.664489186 | 0.112 | 0.289 | 1.27086E-05 |

Abbreviations: Ccl5, C-C motif chemokine ligand 5; Cxcr, CXC chemokine receptor 3; Cd, cluster of differentiation; FC, fold change; Gzmb, granzyme B; Ifng, interferon gamma; Pct, percentage; Pdcd, programmed cell death 1; Prf1, perforin 1; Lag3, lymphocyte-activation gene 3; Tox, thymocyte selection-associated high mobility group box
